# Supplementary figures and images for: Controlled assembly of retinal cells on fractal and Euclidean electrodes
Source: PLoS One. 2022 Apr 6;17(4):e0265685. doi: 10.1371/journal.pone.0265685 (PMC8985931; doi:10.1371/journal.pone.0265685)

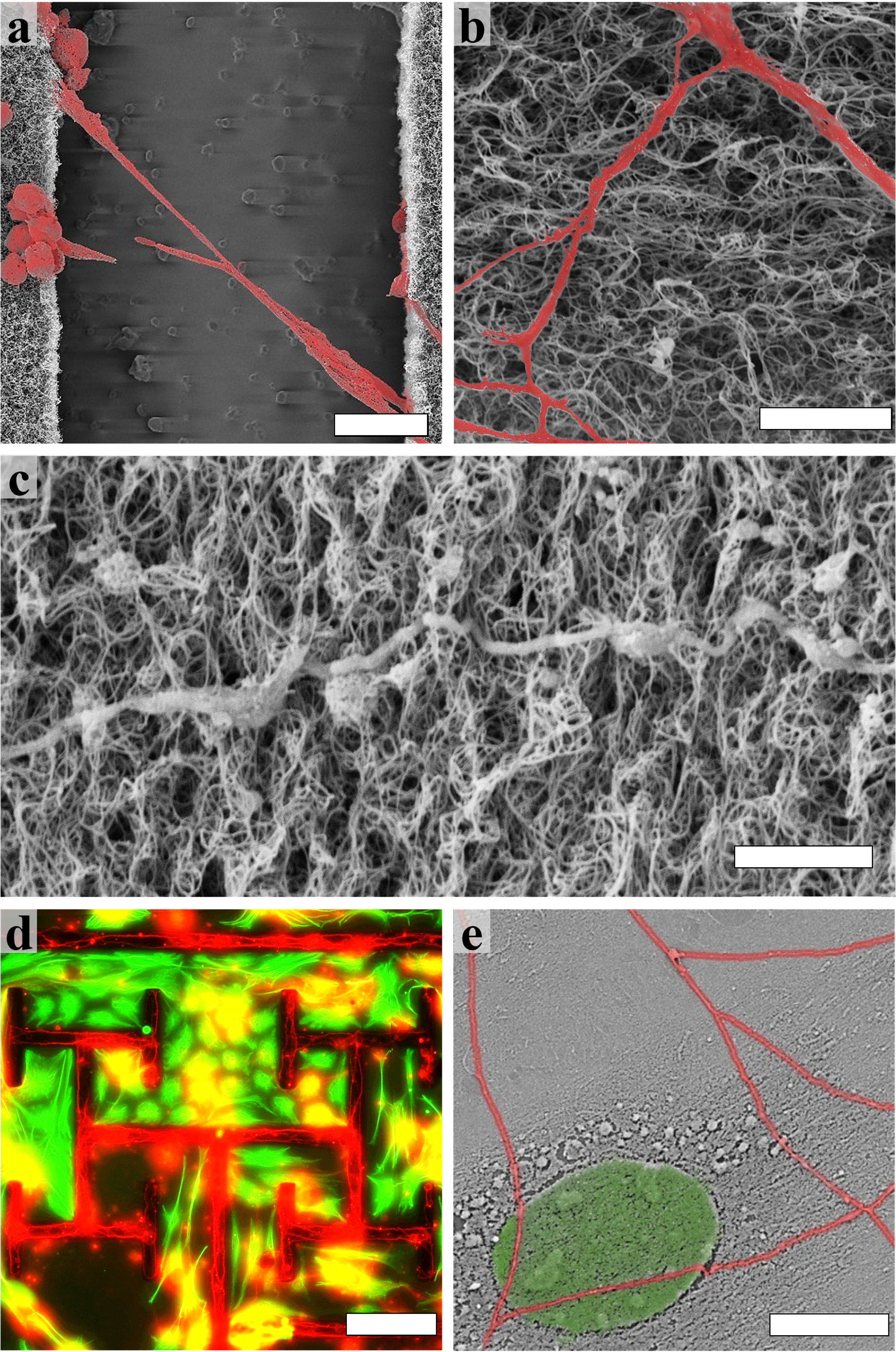

Supplement: S1 Fig — (a) SEM image showing a neuronal process bridging a 50 μm gap between two VACNT rows (7 DIV). (b) SEM image showing neuronal processes on the top surface of a VACNT electrode (7 DIV). (c) SEM image of a neuronal process on the top surface of a VACNT electrode (17 DIV). (d) Merged fluorescence image of a region on a 2–5 fractal showing β-tubulin III labelled neuronal processes (red) attached to and following VACNT branches and GFAP labelled glial cells (green) in the SiO2 gaps (17 DIV). (e) SEM image of a glial cell and neuronal processes on the smooth SiO2 surface (17 DIV). Cell bodies and processes are false-colored in (a), (b), and (e). Scale bars are 10 μm in (a) and (e), 2 μm in (b) and (c), and 75 μm in (d). (TIF) [file pone.0265685.s001.tif]

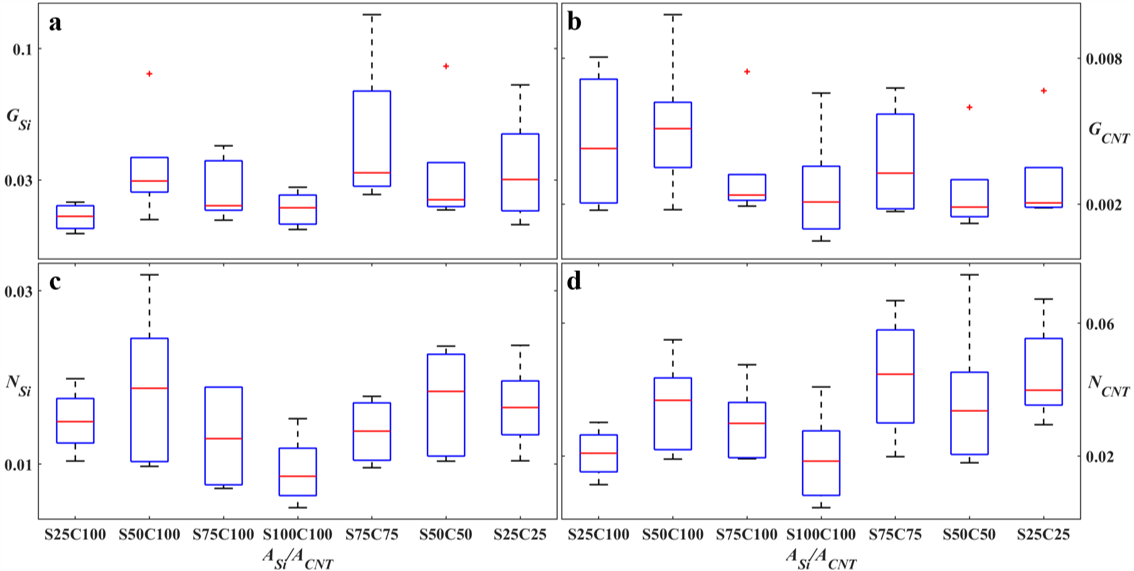

Supplement: S2 Fig — No statistical significance was detected between any pair for all glial and neuronal parameters. (TIF) [file pone.0265685.s002.tif]

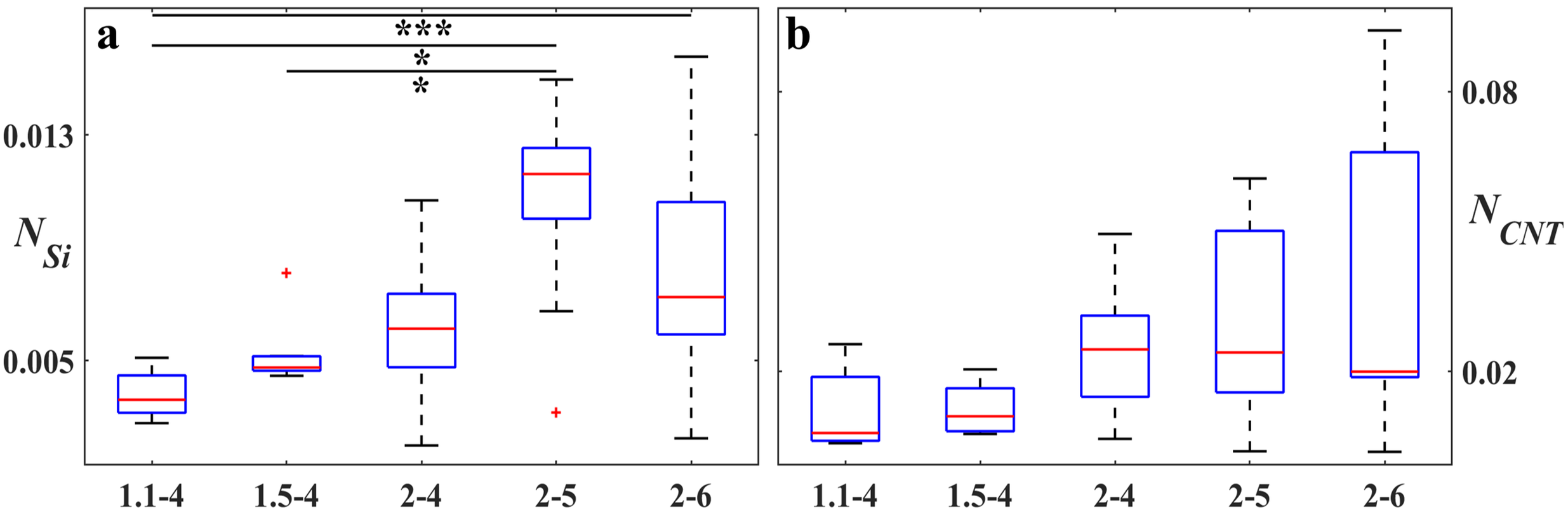

Supplement: S3 Fig — Statistical analysis showing boxplots for NSi (a) and NCNT (b). Stars in (a) indicate the degree of significance: * denotes p ≤ 0.05 and *** denotes p ≤ 0.001. The red plusses in panel (a) are outliers. No significance was observed in NCNT. (TIF) [file pone.0265685.s003.tif]

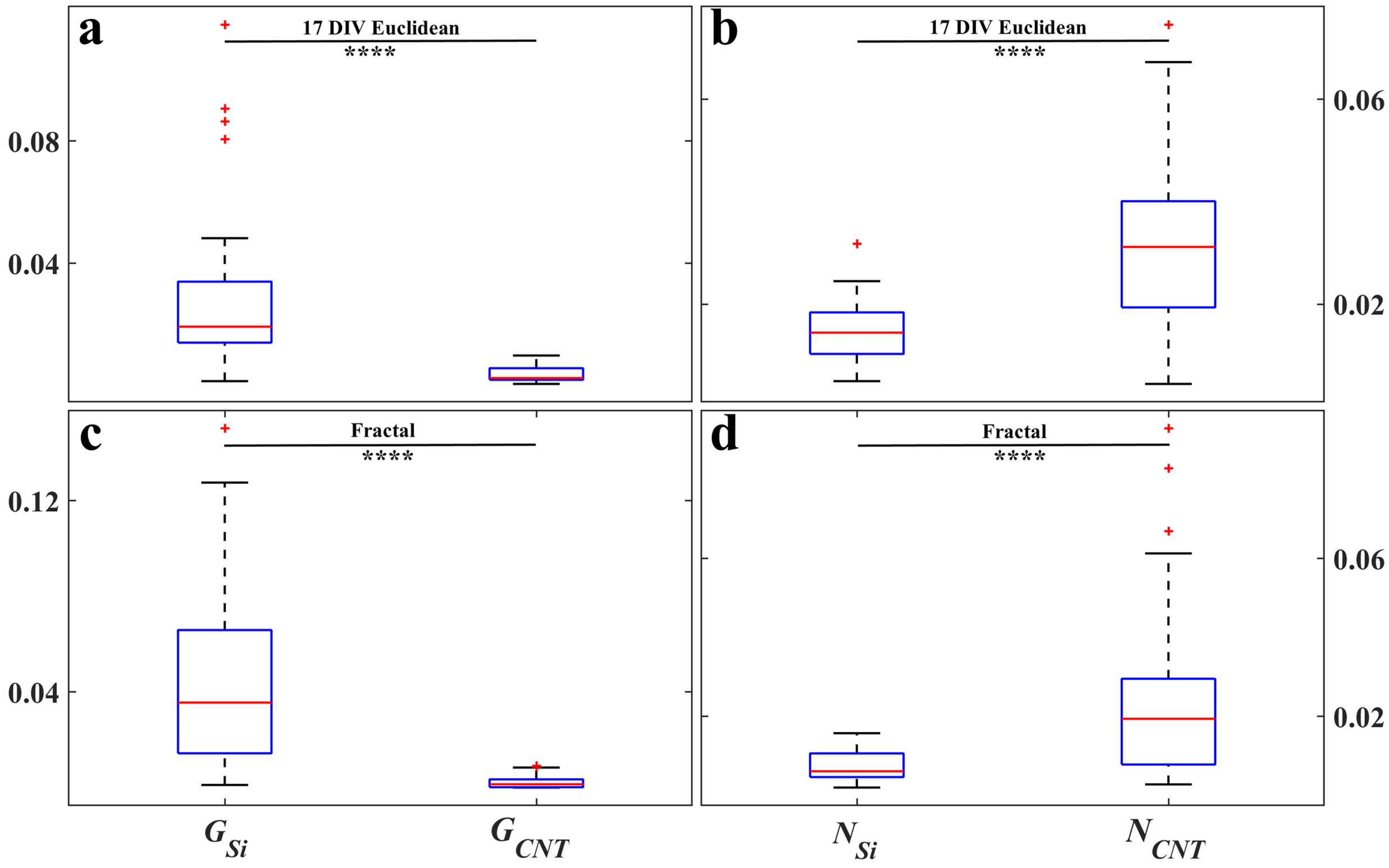

Supplement: S4 Fig — Statistical analysis showing boxplots of GSi vs GCNT for (a) 17 DIV Euclidean and (c) fractals. As well as NSi vs NCNT for (b) 17 DIV Euclidean and (d). Stars in all panels indicate the degree of significance: **** denotes p ≤ 0.0001. The red plusses are outliers. (TIF) [file pone.0265685.s004.tif]

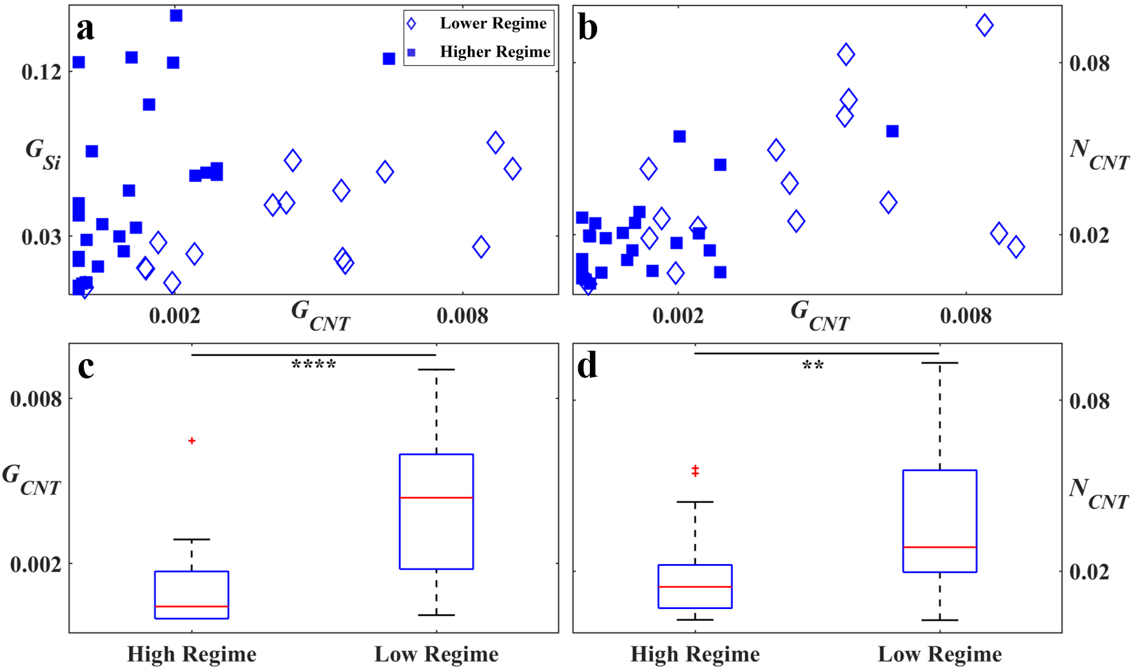

Supplement: S5 Fig — Plots of GSi (a) and NCNT (b) against GCNT showing the different cell behaviors on the VACNT and SiO2 surfaces for the low and high regime fractals. Statistical analysis showing boxplots for GCNT (c) and NCNT (d). No significance was observed in NSi and GSi between the 2 groups. Stars in (c) and (d) indicate the degree of significance: ** denotes p ≤ 0.01 and **** denotes p ≤ 0.0001. The red plusses in panels (c) and (d) for the high regime fractals are outliers. (TIF) [file pone.0265685.s005.tif]
