# Supplementary material for: Controlled assembly of retinal cells on fractal and Euclidean electrodes
Source: PLoS One. 2022 Apr 6;17(4):e0265685. doi: 10.1371/journal.pone.0265685 (PMC8985931; doi:10.1371/journal.pone.0265685)
Supplement: S1 Table — (DOCX) [file pone.0265685.s006.docx]

**S1 Table.** Median values of all glial and neuronal parameters at 3, 7, and 17 DIV for Euclidean and at 17 DIV for fractal electrode types.

| Culture time |  | | 3 DIV | |  | |  |
| --- | --- | --- | --- | --- | --- | --- | --- |
| Samples | *G_Si_* | *G_CNT_* | *N_Si_* | *N_CNT_* | *G* | *N* | *GN* |
| Euclidean electrodes | | | | | | |  |
| S100C100 | 0.0013 | 8.79E-04 | 0.0089 | 0.0217 | 0.6480 | 0.7068 | 0.4479 |
| S75C100 | 0.0014 | 0.0015 | 0.0113 | 0.0386 | 0.4842 | 0.7665 | 0.3557 |
| S50C75 | 0.0043 | 0.0024 | 0.0125 | 0.0275 | 0.6419 | 0.7202 | 0.4677 |
| S50C100 | 0.0047 | 0.0011 | 0.0180 | 0.0462 | 0.7991 | 0.7195 | 0.5718 |
| S25C100 | 0.0036 | 0.0018 | 0.0208 | 0.0629 | 0.6703 | 0.7288 | 0.4934 |
| S50C50 | 0.0037 | 0.0017 | 0.0127 | 0.0252 | 0.6866 | 0.6648 | 0.4568 |
| S25C25 | 0.0024 | 0.0012 | 0.0135 | 0.0230 | 0.6753 | 0.6322 | 0.4473 |
| Culture time |  | | 7 DIV | |  | |  |
| Samples | *G_Si_* | *G_CNT_* | *N_Si_* | *N_CNT_* | *G* | *N* | *GN* |
| Euclidean electrodes | | | | | | |  |
| S100C100 | 0.0065 | 0.0021 | 0.0119 | 0.0494 | 0.7645 | 0.7804 | 0.5872 |
| S75C100 | 0.0101 | 0.0015 | 0.0210 | 0.0511 | 0.8622 | 0.6909 | 0.5915 |
| S50C75 | 0.0134 | 0.0039 | 0.0196 | 0.0573 | 0.8085 | 0.7515 | 0.5685 |
| S50C100 | 0.0106 | 0.0018 | 0.0274 | 0.0475 | 0.8801 | 0.6272 | 0.5374 |
| S25C100 | 0.0054 | 0.0014 | 0.0306 | 0.0500 | 0.7164 | 0.6047 | 0.4650 |
| S50C50 | 0.0053 | 0.0021 | 0.0185 | 0.0493 | 0.8344 | 0.7221 | 0.5649 |
| S25C25 | 0.0105 | 0.0018 | 0.0224 | 0.0587 | 0.8385 | 0.7309 | 0.5984 |
| Culture time |  | | 17 DIV | |  | |  |
| Samples | *G_Si_* | *G_CNT_* | *N_Si_* | *N_CNT_* | *G* | *N* | *GN* |
| Euclidean electrodes | | | | | | |  |
| S100C100 | 0.0152 | 0.0021 | 0.0086 | 0.0185 | 0.8736 | 0.6216 | 0.5500 |
| S75C100 | 0.0163 | 0.0024 | 0.0129 | 0.0298 | 0.8657 | 0.7102 | 0.5901 |
| S50C75 | 0.0338 | 0.0033 | 0.0138 | 0.0446 | 0.9374 | 0.7216 | 0.6833 |
| S50C100 | 0.0294 | 0.0051 | 0.0187 | 0.0368 | 0.8639 | 0.6295 | 0.5286 |
| S25C100 | 0.0106 | 0.0043 | 0.0149 | 0.0208 | 0.6952 | 0.5501 | 0.4015 |
| S50C50 | 0.0194 | 0.0019 | 0.0184 | 0.0336 | 0.9337 | 0.6463 | 0.6033 |
| S25C25 | 0.0302 | 0.0020 | 0.0165 | 0.0398 | 0.9237 | 0.7392 | 0.6515 |
| Fractal electrodes | | | | | | |  |
| 1.1-4 | 0.0187 | 0 | 0.0036 | 0.0068 | 1 | 0.6967 | 0.7049 |
| 1.5-4 | 0.0646 | 0.0015 | 0.0048 | 0.0104 | 0.9845 | 0.6698 | 0.6651 |
| 2-4 | 0.0470 | 0.0016 | 0.0061 | 0.0247 | 0.9566 | 0.7685 | 0.7608 |
| 2-5 | 0.0548 | 0.0011 | 0.0116 | 0.0241 | 0.9724 | 0.6531 | 0.6475 |
| 2-6 | 0.0140 | 0.0014 | 0.0072 | 0.0199 | 0.9190 | 0.7610 | 0.6809 |
