# Supplementary material for: Controlled assembly of retinal cells on fractal and Euclidean electrodes
Source: PLoS One. 2022 Apr 6;17(4):e0265685. doi: 10.1371/journal.pone.0265685 (PMC8985931; doi:10.1371/journal.pone.0265685)
Supplement: Attachment — Submitted filename: PONE-D0-21-36805-Response to reviewers.docx [file pone.0265685.s007.docx]

Dear Dr. Asplund,

We thank the reviewers for their positive reviews of our manuscript, “Controlled Assembly of Retinal Cells on Fractal and Euclidean Electrodes” (PONE-D-21-36805). We have addressed all of their points in the revised manuscript and we believe that their suggestions have improved our submission. We have also addressed all the editorial requirements. Below, we list our revisions in detail. In each case, we have left the reviewer and editorial comments in black font and listed our responses in blue font.

Yours sincerely,

Saba Moslehi, PhD

Post-doctoral Research Fellow

University of Oregon - Department of Physics

[sabam@uoregon.edu](mailto:sabam@uoregon.edu)

**PONE-D-21-36805 reviewer comments-2/7/22. Review Comments to the Author**

**Reviewer #1:** The manuscript entitled “Controlled Assembly of Retinal Cells on Fractal and Euclidean Electrodes” presents an extensive study on how the geometry of electrodes affects the behaviour of retinal cells and glial cells on the surface: their attachment, preferred anchorage places, surface density, process lengths and other parameters that are derived from fractal-like geometry of neurons.

I am impressed with the extensity of research and the amount of valuable results that were derived from a straightforward experimental design. The rationale of this study has been carefully planned, and the results allowed to confirm the initial hypothesis about the effect of fractal geometry on a cell co-culture. I am sure that this study will be an important contribution to the development of biomedical electrodes, not only restricted to interfacing retinal cells, but also other cells and tissues that can be electrically stimulated.

Before accepting the manuscript for publication, I would like Authors to consider the following aspects:

- The introduction section will benefit if the Authors devote a paragraph to the analysis of a geometry of retinal cells. It would be great to cite the research showing that changes in fractal parameters of retinal cells could be associated with cellular processes, such as neurodegeneration (providing that such studies exist).

The reviewer raises a crucial point about the importance of fractality and the use of fractal analysis for studying the morphological characteristics of biological systems especially retinal cells. In response, we have expanded the introduction to cite research that focuses on the characterization of different cell types in the nervous system using fractal analysis. We have separated the references into groups of studies focusing on neurons and glial cells. Additionally, we have categorized them based on the aim of the study, whether it was conducted to distinguish between various subcategories or to diagnose pathological conditions. Given the extensive amount of valuable research conducted in this field, we acknowledge that the list of references added are by no means comprehensive and will serve as a guide to the readers of PLOS One who will be interested in the fractality of biological systems. The added paragraph is now included in the Introduction (lines 87-93).

- A large part of geometrical analysis is focused on retinal cells. I was wondering if glial cells could be also analysed with the use of additional morphometric descriptors. Maybe it would be worth to investigate whether astrocytes could become activated on the electrodes with different geometries? You may check this paper: <https://onlinelibrary.wiley.com/doi/10.1002/glia.22684>

We appreciate the reviewer’s suggestions for studying the morphological characteristics of glial cells to investigate their level of activity in response to the electrode materials and geometry. Such studies are possible following the previously developed methods such as Sholl analysis and fractal box counting methods (e.g., Kang, K. *et al*. “The complex morphology of reactive astrocytes controlled by fibroblast growth factor signaling”, Glia (2014), 62(8), 1328-1344 and Behar, T. “Analysis of Fractal Dimension of O2A Glial Cells Differentiating in Vitro”, Methods (2001), 24(4), 331–9). However, because astrocytes and Müller cells both express glial fibrillary acidic protein (GFAP-marked with immunocytochemistry techniques) in our *in vitro* system, performing the morphology analysis on our current fluorescent images without having the ability to distinguish between the two cell types would complicate interpretations of the results. Because of the importance of such studies for understanding the fundamentals of cell-electrode interactions, we therefore propose future experimental designs that use individual retinal cell markers for all neuron and glial cell types. This will enable us to not only distinguish between the different glial cell types but also between various neurons in our *in vitro* system. Whereas the current studies focused on neuronal process length and glial covering area as the quantifiable measurements of cell morphology and behavior, future studies will focus on more sophisticated morphological characteristics of all cell types. This would not only enable us to determine the glial cell activity level on the electrodes (for Müller cells, astrocytes, and microglia) but also study the effects of their morphological characteristics on the formation of the three ‘boundary’, ‘cluster’, and ‘desert’ regions on the SiO_2_ surface. These suggested future studies are outlined in our future goals in the Conclusions (lines 717-727).

Further studies:

- Detection of anchor points through a focal adhesion analysis.

- Calcium imaging to verify how electrical pulses are transferred between neurons through different electrodes.

We thank the reviewer for suggesting future studies aimed at achieving a better understanding of interactions of cells with the electrode (detection of anchor points) as well as investigating a working electrode (calcium imaging of the electrical activity of the neural network). These suggested future studies are now discussed in the Conclusions (lines 758-766).We propose the use of focal adhesion markers such as vinculin or focal adhesion kinase to detects and analyze the anchor points as well as calcium imaging of the neurons’ electrical activity as part of our future endeavors. These studies will quantify the effects of electrode geometry on the adhesive strength of the neural networks along with their electrical properties.

**Reviewer #2:** The manuscript by Molehi et al. examines an engineered electrode interface with H-tree fractal vs. Euclidian patterned geometries on the behavior of dissociated retinal cell types. The vertical aligned carbon nanotube (VACNT) surfaces appeared to favor the attachment or localization of neurons, whilst the smooth SiO2 surfaces favored glial coverage. Here the authors are using the material properties and surface interactions to 'herd' cells based on their behavior. The fractal parameter of the patterning was therefore investigated to optimize cell-electrode interactions, which were largely quantified morphologically but not functionally verified (ie, by electrophysiology).

The manuscript is fascinating and novel in both its aims and methods, thus worthy of publishing to reach the wide readership of PLoS. It is very well written, although a few minor improvements are suggested below, which should be made to make the results more clear to the reader.

Minor points:

- Figure 3 j1-4 labels are difficult to read as text is too bold. Perhaps this is only in the PDF render, but one might choose another font or not use bold titles. 'Desert' was particularly hard to make out but only after reading the text was it clear what it could be.

We thank the reviewer for their keen observation. All figures have now been edited to meet PLOS One figure requirements and have been tested and approved by the Preflight Analysis and Conversion Engine (PACE) digital diagnostic tool. We hope this resolves the reviewer’s concern on the clarity of the figures.

-please mark statistical significance on Fig3 with asterices or similar to help the reader.

We thank the reviewer for their helpful suggestion for making our results more accessible to the readers. In particular, we appreciate that statistical significances should be included in the figures whenever possible in order to help summarize the details provided in the text. Accordingly, we have now included the standard star notation representation of statistical significance results in Fig. 4 and Fig. 5 and have edited the figure legends to include the interpretation of the stars.

**Editorial requirements:**

1. Please ensure that your manuscript meets PLOS ONE's style requirements, including those for file naming. The PLOS ONE style templates can be found at

<https://journals.plos.org/plosone/s/file?id=wjVg/PLOSOne_formatting_sample_main_body.pdf>

and

<https://journals.plos.org/plosone/s/file?id=ba62/PLOSOne_formatting_sample_title_authors_affiliations.pdf>

We thank the editor for pointing out the style requirements for the manuscript submission. The revised manuscript meets all the PLOS One style requirements.

2. Thank you for stating the following in the Acknowledgments Section of your manuscript:

“All authors participated in the study design; SM, CR, JHS, and WJW created the neuron and glial analysis algorithms; DM and BJA built the CVD system; DM, BJA, SM, and WJW developed the VACNT synthesis process; BJA conceived and implemented the VACNT platform; SM and CR fabricated VACNT electrodes, MTP developed the cell culturing and immunochemistry protocols; 46 SM, CR, JHS, WJW, and MTP performed retinal cell cultures and fluorescence microscopy; SM, CR, and JHS performed image processing and statistical data analysis; SM, CR, JHS, and RPT performed data analysis and validation; SM, CR, JHS, and RPT designed the figures; SM, CR, JHS, MTP, and RPT helped edit figures; SM and RPT drafted the manuscript; RPT coordinated the project; RPT, MTP, BJA, and CMN acquired the funding; all authors edited the manuscript.”

We note that you have provided additional information within the Acknowledgements Section that is not currently declared in your Funding Statement. Please note that funding information should not appear in the Acknowledgments section or other areas of your manuscript. We will only publish funding information present in the Funding Statement section of the online submission form.

Please remove any funding-related text from the manuscript and let us know how you would like to update your Funding Statement.

The added funding statement “RPT, MTP, BJA, and CMN acquired the funding.” is now removed from the Contributions statement. We have updated the Funding Statement as follows:

RPT, CMN, and BJA:

- W. M. Keck Foundation

<http://www.wmkeck.org/>

The funders had no role in study design, data collection and analysis, decision to publish, or preparation of the manuscript.

RPT, WJW, and MTP:

- The Pufendorf Institute

<https://www.pi.lu.se/en/pufendorf-ias>

The funders had no role in study design, data collection and analysis, decision to publish, or preparation of the manuscript.

RPT:

- The Living Legacy Foundation

<https://www.thellf.org/>

The funders had no role in study design, data collection and analysis, decision to publish, or preparation of the manuscript.

- The Ciminelli Foundation

The funders had no role in study design, data collection and analysis, decision to publish, or preparation of the manuscript.

- University of Oregon

<https://www.uoregon.edu/>

The funders had no role in study design, data collection and analysis, decision to publish, or preparation of the manuscript.

MTP:

- The Swedish Research Council - # 2016-03757

<https://www.vr.se/english.html>

The funders had no role in study design, data collection and analysis, decision to publish, or preparation of the manuscript.

- NanoLund at Lund University

<https://www.nano.lu.se/>

The funders had no role in study design, data collection and analysis, decision to publish, or preparation of the manuscript.

- Stiftelsen för Synskadade i f.d. Malmöhus Län

<https://synskadademalmohus.se/>

The funders had no role in study design, data collection and analysis, decision to publish, or preparation of the manuscript.

- Crown Princess Margareta’s Committee for the Blind

The funders had no role in study design, data collection and analysis, decision to publish, or preparation of the manuscript.

3. We note that you have stated that you will provide repository information for your data at acceptance. Should your manuscript be accepted for publication, we will hold it until you provide the relevant accession numbers or DOIs necessary to access your data. If you wish to make changes to your Data Availability statement, please describe these changes in your cover letter and we will update your Data Availability statement to reflect the information you provide.

All the data and the related code is now available at 10.6084/m9.figshare.19127894 and we have updated our Data Availability Statement.

4. Please review your reference list to ensure that it is complete and correct. If you have cited papers that have been retracted, please include the rationale for doing so in the manuscript text, or remove these references and replace them with relevant current references. Any changes to the reference list should be mentioned in the rebuttal letter that accompanies your revised manuscript. If you need to cite a retracted article, indicate the article’s retracted status in the References list and also include a citation and full reference for the retraction notice.

We declare that the reference list is complete and correct and does not include any retracted studies. Upon revising the manuscript the references listed below were added to the list:

44 – 58, 60, 68 – 71, 79, 128, 130, and 131.
